# From Continuous-Flow Mechanical Circulatory Support to Heart Transplantation: Hemodynamic, Immunometabolic, and Body Composition Determinants of Rehabilitation Outcomes

**DOI:** 10.3390/jcm15135305

**Published:** 2026-07-07

**Authors:** Przemysław Lutomski, Krzysztof J. Filipiak, Hanna Wachowiak-Baszyńska, Ewa Straburzyńska-Migaj, Zbigniew Krasiński, Marek Jemielity, Jacek Zieliński, Tomasz Urbanowicz

**Affiliations:** 1 Chair of Physiotherapy, Department of Sport Medicine and Traumatology, Faculty of Health Sciences, Poznan University of Physical Education, 27/39 Królowej Jadwigi Street, 61-871 Poznan, Poland; 2 The Center of Postgraduate Medical Education, 99/103 Marymoncka Street, 01-813 Warsaw, Poland; 3 Cardiac Surgery and Transplantology Department, Poznan University of Medical Sciences, ½ Długa, 61-848 Poznań, Poland; 4 1st Cardiology Department, Poznan University of Medical Sciences, ½ Długa, 61-848 Poznań, Poland; 5 Department of Vascular, Endovascular Surgery, Angiology and Phlebology, Poznan University of Medical Sciences, ½ Dluga Street, 61-848 Poznan, Poland; 6 Department of Athletics, Strength and Conditioning, Poznan University of Physical Education, 27/39 Królowej Jadwigi Street, 61-871 Poznan, Poland

**Keywords:** heart transplantation, left ventricular assist device, rehabilitation, exercise capacity, body composition, sarcopenia, sarcopenic obesity, immunometabolism, endothelial function, pulsatile flow, skeletal muscle, immunosuppression

## Abstract

**Background**: Continuous-flow left ventricular assist devices (LVADs) and heart transplantation (HTX) improve survival and quality of life in advanced heart failure. However, restoration of central hemodynamics does not consistently normalize exercise capacity, physical performance, or body composition. Persistent skeletal muscle dysfunction, endothelial abnormalities, metabolic disturbances, and adverse body composition changes frequently limit functional recovery. **Methods**: This narrative review examines determinants of rehabilitation outcomes across the transition from advanced heart failure to LVAD support and subsequent HTX. Particular emphasis is placed on restoration of pulsatile circulation, vascular and microcirculatory adaptation, immunosuppressive therapy, body composition remodeling, and emerging immunometabolic mechanisms. **Results**: Rehabilitation outcomes appear to be increasingly determined by peripheral rather than central cardiovascular factors. Continuous-flow LVAD support induces vascular, endothelial, autonomic, and microcirculatory adaptations that may persist after transplantation. Although HTX restores physiological pulsatile circulation and cardiac output, recovery is often limited by skeletal muscle dysfunction, impaired mitochondrial capacity, chronotropic abnormalities, and adverse body composition changes. Immunosuppressive therapies further influence muscle plasticity, adipose tissue distribution, insulin sensitivity, endothelial function, and exercise adaptation, contributing to phenotypes such as sarcopenia, myosteatosis, and sarcopenic obesity. **Conclusions**: Functional recovery after LVAD support and HTX is a multidimensional process extending beyond restoration of cardiac function. We propose a hemodynamic–immunometabolic framework in which vascular adaptation, skeletal muscle biology, body composition remodeling, and immunosuppressive therapy interact to determine rehabilitation success and may inform personalized rehabilitation strategies.

## 1. Introduction

Advances in mechanical circulatory support and heart transplantation have transformed the prognosis of patients with advanced heart failure. Continuous-flow left ventricular assist devices (LVADs) have become the dominant strategy for bridge-to-transplantation and durable support, substantially improving survival, end-organ perfusion, and quality of life. Simultaneously, heart transplantation (HTX) remains the gold-standard treatment for eligible patients with end-stage heart failure, offering superior long-term survival and functional recovery [1,2,3]. Despite these therapeutic advances, restoration of central hemodynamics does not invariably translate into normalization of physical performance, exercise capacity, or body composition. Indeed, many patients continue to exhibit persistent skeletal muscle dysfunction, reduced peak oxygen uptake, frailty, and adverse metabolic profiles long after successful transplantation [4].

Traditionally, rehabilitation following LVAD implantation and HTX has been viewed primarily through the lens of cardiac performance and exercise training [5,6,7]. However, accumulating evidence suggests that peripheral determinants of functional capacity may exert a greater influence on long-term outcomes than central cardiac function alone [8,9,10,11]. Skeletal muscle abnormalities, endothelial dysfunction, altered microvascular regulation, chronic inflammation, metabolic derangements, and unfavorable body composition trajectories frequently persist despite correction of the underlying circulatory insufficiency [12,13]. These observations challenge the conventional paradigm that restoration of cardiac output is sufficient to normalize functional status.

An underappreciated aspect of the LVAD-to-transplant continuum is the profound transition in vascular physiology that occurs between continuous-flow mechanical support and transplantation. Continuous-flow LVADs generate a circulation characterized by reduced arterial pulsatility, altered shear stress patterns, impaired baroreceptor signaling, and vascular remodeling [14]. In contrast, HTX restores physiological pulsatile flow and normalizes many aspects of macrovascular hemodynamics [15]. Yet peripheral tissues exposed to years of advanced heart failure and prolonged non-pulsatile support may undergo structural and metabolic adaptations that are not immediately reversible [16,17]. Consequently, the transition from LVAD support to transplantation represents not merely a change in cardiac replacement therapy but a fundamental shift in systemic vascular biology.

Simultaneously, transplantation introduces an entirely different physiological challenge: lifelong immunosuppressive therapy. While immunosuppressive regimens are indispensable for preventing allograft rejection, they exert profound effects on skeletal muscle metabolism, adipose tissue distribution, insulin sensitivity, mitochondrial function, and exercise adaptation [18,19,20,21,22,23]. Thus, the post-transplant recovery trajectory emerges from the interaction between restored hemodynamics and pharmacologically modified metabolism.

Importantly, body composition has emerged as a central yet insufficiently studied determinant of outcomes across the LVAD-transplant pathway. Patients with advanced heart failure often present with cardiac cachexia, sarcopenia, or severe muscle wasting [24] Although LVAD implantation frequently reverses catabolic processes and improves nutritional status, gains in adipose tissue often exceed recovery of lean body mass [25]. Following transplantation, additional weight gain is common, frequently driven by immunosuppressive therapy, reduced physical activity, and metabolic dysregulation [26]. Consequently, many patients transition from cachexia to sarcopenic obesity, a phenotype characterized by excess adiposity coexisting with impaired muscle quality and function. This phenotype may adversely affect exercise tolerance, cardiometabolic health, and long-term transplant outcomes despite apparently successful restoration of cardiac function.

Recent advances in immunometabolism provide a framework for integrating these observations [27,28]. Likewise, skeletal muscle serves as a major regulator of systemic metabolism by producing myokines and maintaining glucose homeostasis [29].

We propose that rehabilitation outcomes following LVAD support and subsequent heart transplantation are governed by three interconnected biological domains:

(1) Hemodynamic adaptation to the transition from continuous-flow to pulsatile circulation;
(2) Immunosuppressive modulation of skeletal muscle and adipose tissue metabolism; and
(3) Dynamic changes in body composition and peripheral tissue function.

Rather than viewing rehabilitation as a consequence of improved cardiac output alone, we suggest that recovery should be conceptualized as a multidimensional process involving vascular, metabolic, immune, and musculoskeletal adaptation.

This review examines the physiological consequences of continuous-flow and pulsatile circulatory states, explores the impact of immunosuppressive therapy on exercise responsiveness and body composition, and discusses the emerging role of immunometabolic mechanisms in determining rehabilitation outcomes. By integrating these traditionally separate fields, we propose a novel hemodynamic-immunometabolic framework that may better explain the heterogeneity of functional recovery observed in patients transitioning from LVAD support to HTX.

To facilitate understanding of the complex physiological transitions occurring throughout the heart failure–LVAD–HTX continuum, the major hemodynamic, vascular, metabolic, and body composition characteristics associated with each stage are summarized in Table 1. Importantly, the table highlights that although central circulatory function progressively improves across the continuum, normalization of peripheral physiology remains incomplete, providing a conceptual framework for the persistent functional limitations observed in many transplant recipients.

**Figure 1:**
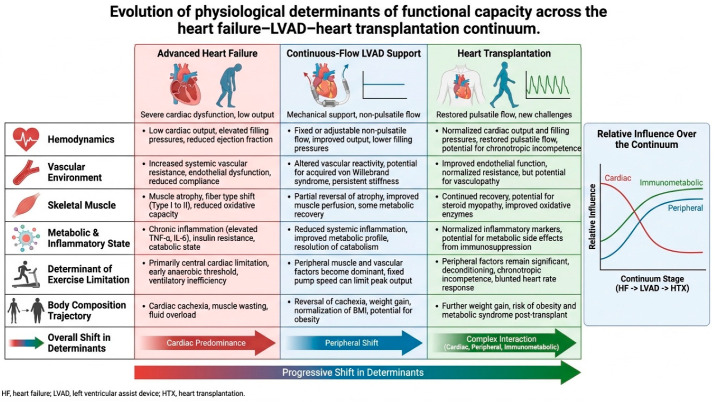
Evolution of physiological determinants of functional capacity across the heart failure–LVAD–heart transplantation continuum. (Created with FigureLabs by T. Urbanowicz (ID: FL-PUB-20260611-I5KKDE). https://www.figurelabs.ai/ (accessed on 10 June 2026)).

Understanding this physiological shift is essential for explaining why normalization of cardiac function does not invariably result in normalization of exercise capacity (Figure 1).

### 1.1. Methodology

A structured literature search was performed in PubMed/MEDLINE covering publications from January 2000 to March 2026. Search terms included combinations of: ‘heart transplantation’, ‘LVAD’, ‘left ventricular assist device’, ‘continuous-flow’, ‘pulsatile flow’, ‘rehabilitation’, ‘exercise capacity’, ‘VO_2_peak’, ‘endothelial function’, ‘skeletal muscle’, ‘sarcopenia’, ‘myosteatosis’, ‘body composition’, ‘immunometabolism’, and ‘immunosuppression’, together with corresponding MeSH terms. Priority was given to original studies, clinical trials, observational cohorts, systematic reviews, consensus statements, and guideline documents published in English. Studies unrelated to rehabilitation, exercise physiology, body composition, vascular adaptation, or transplant outcomes were excluded. Literature screening and article selection were independently performed by two authors, with disagreements resolved through discussion. Because of the narrative nature of this review, formal PRISMA methodology was not applied.

Priority was given to original research articles, clinical trials, observational studies, systematic reviews, and position papers published in English. Additional references were identified through manual review of the bibliographies of relevant articles. The selected literature was evaluated for its relevance to the physiological and clinical determinants of rehabilitation outcomes during the transition from advanced heart failure to LVAD support and subsequent heart transplantation. Where heart-transplant-specific evidence was unavailable, selected data from kidney, liver, and mixed solid-organ transplant cohorts were included to provide a mechanistic context. Such extrapolation is explicitly acknowledged, and conclusions drawn from non-cardiac transplant populations should be interpreted with caution.

Despite increasing recognition of peripheral determinants of functional capacity after LVAD implantation and HTX, the available literature remains fragmented. Vascular adaptation, skeletal muscle biology, body composition remodeling, immunometabolism, and immunosuppressive therapy are typically discussed as separate domains, despite substantial physiological overlap. Consequently, a comprehensive framework that integrates these processes into a unified model of rehabilitation outcomes remains lacking.

The purpose of the present review is therefore threefold: (i) to summarize current evidence regarding vascular and peripheral adaptation across the advanced heart failure–LVAD–HTX continuum; (ii) to examine the influence of body composition remodeling and immunosuppressive therapy on functional recovery; and (iii) to explore how these interrelated processes may collectively determine rehabilitation responsiveness after transplantation. Based on this synthesis, we subsequently propose an integrative hemodynamic–immunometabolic framework intended to generate future mechanistic and clinical research hypotheses.

### 1.2. Relationship to Existing Consensus Documents and Reviews

Various authoritative documents have addressed rehabilitation following HTX and LVAD implantation [4,53,54,55]. Current ISHLT recommendations emphasize exercise training as a cornerstone of post-transplant management, whereas the EAPC/HFA/ESOT consensus statement highlights the importance of lifelong cardiovascular prevention, the promotion of physical activity, and multidisciplinary rehabilitation [4]. Similarly, systematic reviews and Cochrane analyses have consistently demonstrated the safety of exercise-based rehabilitation after HTX and its beneficial effects on peak oxygen uptake, exercise capacity, and quality of life [56]. Contemporary evidence further supports the broad role of cardiac rehabilitation across cardiovascular populations, including patients with atrial fibrillation, where structured rehabilitation programs improve functional capacity, symptom burden, and overall cardiovascular health [57].

Nevertheless, these documents primarily focus on rehabilitation efficacy and clinical implementation. In contrast, substantially less attention has been devoted to the biological determinants responsible for the marked heterogeneity in rehabilitation responsiveness observed among patients with apparently similar graft function and exercise exposure.

The present review, therefore, does not attempt to re-establish the benefits of rehabilitation. Rather, it focuses on the mechanistic interfaces between vascular adaptation, skeletal muscle biology, body composition remodeling, immunometabolism, and immunosuppressive therapy, with particular attention to the physiological transition from continuous-flow mechanical circulatory support to restored pulsatile circulation following transplantation.

Most available publications primarily focus on exercise safety, training prescription, clinical outcomes, and prognostic implications. In contrast, substantially less attention has been devoted to the interactions among vascular adaptation, skeletal muscle remodeling, body composition trajectories, immunometabolism, and immunosuppressive therapy across the entire heart failure–LVAD–HTX continuum. To contextualize the rationale for the present review, Table 2 summarizes key areas of established evidence and guideline recommendations while highlighting the specific conceptual and mechanistic perspectives explored herein.

## 2. The Physiological Significance of Pulsatile Blood Flow

Pulsatile blood flow represents a fundamental characteristic of normal cardiovascular physiology. Beyond its role in generating systemic perfusion, pulsatility serves as a critical biological signal influencing endothelial function, vascular tone, microcirculatory regulation, organ perfusion, and tissue metabolism [58]. Cyclic changes in pressure and flow produce oscillatory shear forces that are sensed by endothelial mechanoreceptors, triggering intracellular signaling pathways that regulate nitric oxide production, vascular remodeling, angiogenesis, and anti-inflammatory responses [59,60]. Consequently, the physiological importance of pulsatility extends far beyond simple blood transport and encompasses complex interactions between the cardiovascular system and peripheral tissues.

The advent of continuous-flow LVAD technology fundamentally altered this physiological paradigm. Modern continuous-flow devices generate near-continuous blood flow with markedly reduced pulse pressure and diminished pulsatile energy transmission throughout the arterial tree [61,62]. Although these devices successfully restore systemic perfusion and improve survival, they simultaneously expose the vasculature and peripheral tissues to an artificial hemodynamic environment that differs substantially from normal physiology.

The long-term consequences of this altered flow pattern remain incompletely understood. However, growing evidence suggests that continuous-flow support induces widespread vascular, endothelial, and microcirculatory adaptations that may contribute to persistent functional limitations despite normalization of central hemodynamics [63].

Although continuous-flow LVADs successfully restore systemic perfusion, they expose the vascular system to a markedly different mechanical environment from that of physiological circulation [64]. Restoration of pulsatility following HTX may therefore represent a critical biological stimulus for vascular and metabolic recovery. Summary of major vascular, endothelial, autonomic, and microcirculatory adaptations associated with continuous-flow support and the potential physiological benefits associated with restoration of pulsatile circulation after transplantation. Proposed implications for exercise capacity and rehabilitation outcomes are summarized in Table 3.

## 3. Vascular Adaptation During Continuous-Flow Support

The vascular system functions as a dynamic organ continuously responding to mechanical stimuli. Under physiological conditions, pulsatile flow regulates endothelial homeostasis through shear stress-dependent activation of multiple signaling pathways, including endothelial nitric oxide synthase, vascular endothelial growth factor signaling, and anti-inflammatory transcriptional programs.

Continuous-flow support substantially modifies these stimuli. Experimental and clinical studies have demonstrated reductions in endothelial nitric oxide bioavailability, alterations in vascular smooth muscle function, and structural changes within the arterial wall during prolonged exposure to diminished pulsatility [74,75]. Reduced cyclic stretch may promote arterial stiffening, impair vasomotor responsiveness, and alter vascular compliance.

Importantly, these changes occur despite improvements in systemic perfusion. Consequently, restoration of cardiac output does not necessarily translate into normalization of peripheral vascular function. This phenomenon may help explain why exercise capacity frequently remains impaired after LVAD implantation despite dramatic improvements in resting hemodynamics.

Baroreceptor physiology may also be affected. Chronic reduction in pulse pressure attenuates physiological stimulation of arterial baroreceptors, potentially contributing to autonomic dysregulation and impaired cardiovascular responsiveness during exercise [37,76]. Altered sympathetic–parasympathetic balance has been reported in LVAD recipients and may persist despite successful mechanical unloading of the failing ventricle.

Collectively, these observations suggest that continuous-flow support creates a unique vascular phenotype characterized by altered mechanotransduction, endothelial dysfunction, and impaired adaptive responsiveness.

## 4. Microcirculation and Skeletal Muscle Perfusion

While macrovascular hemodynamics receive considerable attention in LVAD research, the microcirculation represents the principal site of oxygen and nutrient exchange. Exercise performance depends largely on the ability of skeletal muscle microvasculature to recruit capillaries, redistribute blood flow, and match oxygen delivery to metabolic demand [77,78].

Advanced heart failure is associated with profound microvascular abnormalities, including capillary rarefaction, endothelial dysfunction, impaired vasodilatory reserve, and reduced skeletal muscle perfusion. Although LVAD implantation improves systemic circulation, several studies suggest that microvascular recovery remains incomplete.

Reduced pulsatility may contribute to persistent abnormalities in capillary recruitment and tissue oxygen extraction [79]. Furthermore, chronic exposure to non-physiological flow patterns may alter local endothelial signaling pathways involved in angiogenesis and vascular remodeling [80].

These findings are particularly relevant because peripheral oxygen utilization rather than cardiac output often becomes the primary determinant of exercise limitation following LVAD implantation. Improvements in central circulation may therefore reveal previously masked peripheral dysfunction rather than fully correcting it.

## 5. Heart Transplantation: Restoration of Pulsatile Physiology

Heart transplantation re-establishes physiological pulsatile circulation and restores many of the hemodynamic characteristics absent during continuous-flow support. Pulse pressure normalizes, arterial stretch is reintroduced, and endothelial cells are once again exposed to cyclical mechanical stimuli.

The restoration of pulsatility may represent an important but underrecognized mechanism contributing to post-transplant functional recovery. Experimental data suggest that pulsatile flow promotes endothelial nitric oxide production, enhances microvascular responsiveness, improves tissue perfusion, and supports physiological vascular remodeling. In theory, these changes should facilitate recovery of peripheral tissues previously exposed to prolonged heart failure and mechanical support.

However, restoration of pulsatility does not immediately reverse years of vascular and metabolic adaptation. Similar to the concept of cardiac remodeling, peripheral tissues may exhibit a form of “hemodynamic memory” whereby structural and molecular alterations persist despite normalization of circulatory conditions.

Indeed, many transplant recipients continue to demonstrate impaired endothelial function, reduced exercise capacity, and skeletal muscle abnormalities despite excellent graft function [81,82]. These observations suggest that recovery of peripheral physiology may lag substantially behind restoration of central hemodynamics.

### 5.1. Cardiac Denervation and Chronotropic Limitation

Although transplantation restores physiological pulsatile circulation and normalizes cardiac output, it does not fully restore autonomic regulation of the heart. Surgical transplantation results in functional cardiac denervation, producing characteristic alterations in heart rate control that may significantly influence exercise performance [47,83].

Resting heart rate is typically elevated, whereas heart rate responsiveness during exercise is delayed and frequently blunted. The absence of immediate autonomic modulation results in reliance on circulating catecholamines to increase cardiac output during physical exertion [84,85]. Consequently, transplant recipients often exhibit chronotropic incompetence, delayed achievement of peak heart rate, and prolonged recovery following exercise.

These abnormalities may contribute substantially to persistent reductions in peak oxygen uptake despite preserved graft function. Importantly, chronotropic limitation coexists with peripheral abnormalities involving skeletal muscle, endothelium, and body composition, creating a multifactorial basis for exercise intolerance [86].

Partial autonomic reinnervation may occur in selected recipients over time, although the extent and functional significance of this process remain highly variable. Consequently, exercise capacity after transplantation reflects not only recovery of pulsatile circulation but also the complex interaction between autonomic adaptation and peripheral tissue remodeling.

Recognition of chronotropic limitation is particularly important when designing rehabilitation programs, as conventional exercise prescriptions based on heart rate may inadequately reflect physiological effort in transplant recipients [87].

### 5.2. The LVAD-to-HTX Transition as a Model of Vascular Adaptation

The bridge-to-transplant patient represents a unique human model for studying vascular adaptation. Few clinical scenarios involve prolonged exposure to markedly reduced pulsatility followed by the abrupt restoration of physiological blood flow.

Despite the clinical relevance of this transition, rehabilitation studies have largely focused on improvements in peak oxygen consumption, walking distance, and quality-of-life measures while giving relatively little attention to underlying vascular biology [88,89]. As a result, the potential contribution of restored pulsatility to rehabilitation outcomes remains poorly defined.

## 6. A Hemodynamic Foundation for the Immunometabolic Model

The restoration of pulsatile circulation following transplantation may provide a permissive environment for recovery, but the ultimate extent of rehabilitation likely depends on concurrent metabolic and immunological factors [50,90,91]. This concept forms the basis of a broader hemodynamic-immunometabolic model in which vascular recovery interacts with body composition remodeling and immunosuppressive therapy to determine long-term functional outcomes.

Understanding these interactions may be essential for developing personalized rehabilitation strategies to optimize recovery in the growing population of patients transitioning from LVAD support to HTX.

### Hemodynamic Memory: A Proposed Hypothesis for Persistent Peripheral Adaptation After Continuous-Flow Support

Importantly, the concept of hemodynamic memory should presently be regarded as a hypothesis-generating framework rather than an established biological mechanism. Although persistent endothelial, skeletal muscle, mitochondrial, and microvascular abnormalities have been documented following successful transplantation, direct evidence linking these findings to prior exposure to continuous-flow support remains limited. The proposed model is therefore intended to provide a conceptual structure for future mechanistic investigation rather than a definitive explanation of post-transplant functional recovery.

The concept of biological memory has gained increasing recognition across multiple areas of cardiovascular medicine. Metabolic memory in diabetes and inflammatory memory in chronic immune-mediated diseases illustrate how transient physiological exposures can induce long-lasting cellular- and tissue-level adaptations that persist even after the initiating stimulus is removed. A similar phenomenon may occur in patients supported with continuous-flow LVADs.

Despite restoration of physiological pulsatility following HTX, recovery of peripheral function is frequently incomplete. Endothelial dysfunction, impaired skeletal muscle oxidative capacity, reduced exercise tolerance, and abnormal body composition may persist despite normalization of cardiac output and graft function. These observations raise the possibility that prolonged exposure to continuous-flow physiology may induce a persistent biological signature that is not immediately reversible.

Continuous-flow support modifies several fundamental determinants of vascular homeostasis, including shear stress patterns, cyclic arterial stretch, endothelial mechanotransduction, autonomic regulation, and microvascular perfusion. Chronic exposure to these altered stimuli may promote structural and molecular adaptations within endothelial cells, vascular smooth muscle, skeletal muscle, and mitochondria. Such adaptations could persist after transplantation and influence subsequent responses to exercise training and rehabilitation.

This concept may be described as hemodynamic memory, defined as the persistence of flow-dependent biological adaptations despite restoration of physiological circulation. Similar to metabolic memory, hemodynamic memory may operate through multiple mechanisms, including epigenetic modification, altered endothelial signaling, mitochondrial remodeling, microvascular rarefaction, and long-lasting changes in tissue metabolism.

The concept of hemodynamic memory remains largely speculative but provides a potentially important framework for understanding the marked heterogeneity observed in post-transplant recovery. Patients exposed to prolonged periods of continuous-flow support may enter transplantation with differing degrees of vascular and skeletal muscle remodeling, resulting in variable rehabilitation trajectories despite similar graft function.

Future studies investigating endothelial phenotype, skeletal muscle transcriptomics, mitochondrial function, and vascular responsiveness before and after transplantation may help determine whether hemodynamic memory represents a measurable biological phenomenon and whether it contributes to long-term functional outcomes.

**Figure 2:**
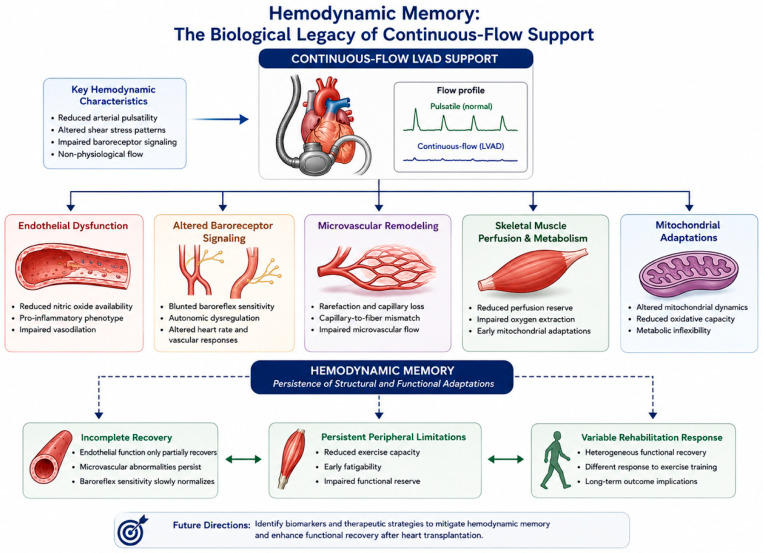
Proposed concept of hemodynamic memory following prolonged continuous-flow mechanical circulatory support. (Created with FigureLabs, by T. Urbanowicz (FL-PUB-20260611-R2JXGE). https://www.figurelabs.ai/ (accessed on 10 June 2026)).

Continuous-flow LVAD support exposes the vascular system and peripheral tissues to a markedly different mechanical environment characterized by diminished pulsatility, altered shear stress patterns, and impaired mechanotransduction. We propose that prolonged exposure to these conditions may induce persistent biological adaptations that continue to influence functional recovery even after restoration of physiological circulation following transplantation, as shown in Figure 2.

## 7. Skeletal Muscle in Advanced Heart Failure: The Catabolic State

Advanced heart failure is characterized by profound metabolic dysregulation resulting from chronic neurohormonal activation, systemic inflammation, reduced tissue perfusion, and physical inactivity [92,93]. Together, these factors create a persistent catabolic environment promoting skeletal muscle wasting and functional decline.

At the cellular level, heart failure is associated with activation of proteolytic pathways, including the ubiquitin–proteasome system, suppression of anabolic signaling, mitochondrial dysfunction, and impaired oxidative phosphorylation. Histological studies have demonstrated reductions in muscle fiber cross-sectional area, preferential atrophy of type I oxidative fibers, decreased capillary density, and diminished mitochondrial content.

These abnormalities contribute directly to impaired oxygen extraction and reduced exercise tolerance. Importantly, the resulting phenotype extends beyond simple loss of muscle mass. Muscle quality, metabolic flexibility, and contractile efficiency are simultaneously compromised, producing a complex syndrome that overlaps with frailty, sarcopenia, and cardiac cachexia.

Cardiac cachexia represents the most severe manifestation of this process and is associated with increased mortality independent of cardiac function. Characterized by involuntary weight loss, loss of skeletal muscle, adipose tissue depletion, and systemic inflammation, cachexia reflects a profound disturbance in whole-body energy homeostasis. Many patients ultimately receiving LVAD support or transplantation enter treatment with significant pre-existing deficits in muscle mass and physical reserve.

### Acute Mechanical Circulatory Support as an Intermediate Biological State

Not all patients progress to durable LVAD support or HTX through a stable chronic heart failure trajectory. A significant proportion require temporary mechanical circulatory support, including venoarterial extracorporeal membrane oxygenation (VA-ECMO), intra-aortic balloon pump (IABP), or Impella devices as bridge-to-decision, bridge-to-LVAD, or bridge-to-transplant strategies [94,95,96]. These patients are exposed to profound inflammatory activation, critical illness, immobilization, skeletal muscle wasting, nutritional deficits, and accelerated deconditioning. Consequently, pre-transplant body composition and rehabilitation potential may be strongly influenced by the duration and severity of temporary circulatory support. These factors may contribute to subsequent heterogeneity of post-transplant recovery and represent an important extension of the hemodynamic–immunometabolic framework proposed in this review.

## 8. LVAD Support: Reversal of Catabolism or Emergence of a New Phenotype?

Mechanical circulatory support dramatically improves systemic perfusion, reduces neurohormonal activation, and alleviates many drivers of catabolism. Consequently, LVAD implantation frequently results in weight gain, improved appetite, enhanced nutritional status, and partial restoration of physical activity.

However, emerging data suggest that recovery of body composition may not be physiologically balanced. In many patients, gains in adipose tissue exceed recovery of skeletal muscle mass. While total body weight increases, improvements in lean tissue are often modest and incomplete. Consequently, apparent nutritional recovery may obscure persistent deficits in muscle quantity and quality.

Several mechanisms may contribute to this phenomenon. Prolonged preoperative deconditioning, persistent inflammation, endocrine abnormalities, and limited exercise capacity may continue to impair muscle anabolism despite improvements in hemodynamics. Additionally, exposure to continuous-flow physiology may influence microvascular function and skeletal muscle perfusion, potentially limiting complete restoration of peripheral tissue health.

## 9. Heart Transplantation and the Paradox of Functional Recovery

HTX removes many of the hemodynamic constraints associated with advanced heart failure and mechanical support. Restoration of physiological cardiac output, improved organ perfusion, and enhanced exercise tolerance create conditions theoretically favorable for complete musculoskeletal recovery.

Yet numerous studies have demonstrated that skeletal muscle abnormalities persist long after transplantation. Reduced peak oxygen uptake, diminished muscle strength, impaired mitochondrial function, and abnormalities in body composition remain evident even among recipients with excellent graft function.

Muscle biopsies obtained after transplantation have demonstrated persistent reductions in oxidative capacity, abnormalities in mitochondrial structure, and incomplete reversal of fiber-type alterations. Furthermore, improvements in exercise performance often correlate more closely with peripheral adaptations than with indices of cardiac function, emphasizing the importance of skeletal muscle as a primary determinant of rehabilitation success.

## 10. Definitions and Clinical Assessment of Body Composition Phenotypes

### 10.1. Sarcopenia

Sarcopenia is a progressive skeletal muscle disorder characterized by loss of muscle strength, mass, and function. The revised European Working Group on Sarcopenia in Older People (EWGSOP2) emphasizes low muscle strength as the primary diagnostic criterion, recognizing it as the most reliable predictor of adverse clinical outcomes [97]. A diagnosis of sarcopenia is confirmed when reduced muscle strength is accompanied by evidence of diminished muscle quantity or quality, while impaired physical performance indicates severe sarcopenia. The condition is associated with increased risks of falls, frailty, disability, hospitalization, and mortality, particularly among older adults and individuals with chronic diseases.

### 10.2. Myosteatosis

Myosteatosis refers to the pathological infiltration and accumulation of adipose tissue within and between skeletal muscle fibers [98]. Unlike sarcopenia, which primarily involves a reduction in muscle mass and strength, myosteatosis reflects impaired muscle composition and quality despite the potential preservation of overall muscle quantity. Excess intramuscular fat impairs muscle metabolism, contractile function, and insulin sensitivity, thereby contributing to reduced physical performance and adverse clinical outcomes. Increasing evidence suggests that myosteatosis represents a distinct component of muscle dysfunction and may independently predict morbidity and mortality across a range of patient populations.

### 10.3. Sarcopenic Obesity

Sarcopenic obesity [99,100] is a complex body-composition phenotype characterized by the coexistence of excess adiposity and reduced muscle strength and/or mass. This condition combines the adverse metabolic consequences of obesity with the functional impairments associated with sarcopenia, resulting in a synergistic increase in health risks. Individuals with sarcopenic obesity often experience greater limitations in physical function, heightened systemic inflammation, insulin resistance, and poorer clinical outcomes compared with those presenting with either obesity or sarcopenia alone. Recognition of this phenotype is increasingly important in both clinical practice and research, as conventional measures such as body mass index may fail to identify the underlying loss of muscle health.

### 10.4. Visceral Obesity

Visceral obesity [101,102] is characterized by excessive accumulation of adipose tissue within the abdominal cavity, surrounding internal organs. In contrast to subcutaneous fat, visceral adipose tissue is highly metabolically active and contributes substantially to systemic inflammation, insulin resistance, and cardiometabolic dysfunction. A growing body of evidence identifies visceral obesity as a major driver of metabolic syndrome, type 2 diabetes, cardiovascular disease, and several obesity-related malignancies.

### 10.5. Recommended Assessment Modalities

Accurate characterization of sarcopenia, myosteatosis, visceral obesity, and sarcopenic obesity requires the use of validated body composition assessment techniques. Dual-energy X-ray absorptiometry (DXA), computed tomography (CT), magnetic resonance imaging (MRI), and bioelectrical impedance analysis (BIA) are commonly used to evaluate sarcopenia, with DXA, CT, and MRI providing the most robust estimates of muscle mass. Myosteatosis is best assessed using CT-derived muscle attenuation measurements or advanced MRI techniques that quantify intramuscular fat infiltration. Visceral obesity is most accurately assessed with CT and MRI, whereas DXA offers a practical alternative for estimating central adiposity. The diagnosis of sarcopenic obesity requires an integrated approach combining body composition assessment with objective measures of muscle strength and physical performance. The preferred assessment tools for the described phenotypes are shown in Table 4.

## 11. Body Composition Remodeling After Transplantation

Weight gain following HTX is common and often substantial. Although some degree of weight restoration constitutes recovery from chronic illness, excessive adipose tissue accumulation has become an increasingly important clinical problem.

Several factors contribute to post-transplant weight gain. Improved appetite, resolution of advanced heart failure, reduced catabolic signaling, and decreased symptom burden promote positive energy balance. Simultaneously, physical activity frequently remains below recommended levels despite successful transplantation.

The introduction of immunosuppressive therapy further amplifies these effects. Corticosteroids promote adipogenesis, increase appetite, induce insulin resistance, and favor central fat deposition. Calcineurin inhibitors contribute to metabolic disturbances and hypertension, whereas mTOR inhibitors may influence nutrient sensing and energy metabolism through direct effects on cellular growth pathways.

Consequently, many transplant recipients develop excess adiposity, particularly within visceral compartments. This pattern is clinically significant because visceral adipose tissue functions as an active endocrine organ, producing inflammatory cytokines, adipokines, and metabolic mediators that influence cardiovascular risk and physical performance.

The resulting phenotype is often characterized not simply by obesity but by a coexistence of increased fat mass and impaired muscle function—a condition increasingly recognized as sarcopenic obesity.

### 11.1. Sarcopenic Obesity: The Emerging Post-Transplant Phenotype

Sarcopenic obesity represents the convergence of two adverse processes: progressive adipose tissue accumulation and inadequate preservation of skeletal muscle mass and function [113]. Although traditionally viewed as distinct entities, obesity and sarcopenia frequently coexist and may interact synergistically to impair physical performance.

In transplant recipients, this phenotype may be particularly relevant. Excess adiposity increases inflammatory signaling, promotes insulin resistance, and impairs physical mobility. Simultaneously, muscle weakness reduces exercise capacity and limits the ability to engage in effective rehabilitation.

Importantly, body mass index frequently fails to identify these abnormalities. Two transplant recipients with identical body mass indices may have markedly different proportions of lean and adipose tissue and, therefore, markedly different metabolic and functional profiles. Reliance on body weight alone may therefore underestimate the burden of adverse body composition following transplantation.

Emerging evidence suggests that measures of muscle mass, muscle quality, and visceral adiposity may provide superior prognostic information compared with conventional anthropometric indices. These observations support a growing shift from weight-centered assessments toward comprehensive body composition phenotyping in transplant populations.

### 11.2. Skeletal Muscle as an Immunometabolic Organ

Traditionally, skeletal muscle has been viewed primarily as a locomotor tissue. Contemporary research, however, increasingly recognizes skeletal muscle as a major endocrine and immunometabolic organ [114].

## 12. Immunosuppressive Therapy as a Determinant of Muscle Plasticity, Body Composition, and Exercise Adaptation

### 12.1. Rehabilitation in the Era of Immunosuppression

The remarkable improvement in survival achieved through modern immunosuppressive strategies has fundamentally altered the landscape of HTX. Contemporary therapeutic regimens have substantially reduced acute rejection and improved long-term graft survival, transforming HTX into a chronic disease model requiring lifelong pharmacological immune modulation. While the cardiovascular and immunological consequences of immunosuppression have been extensively investigated, considerably less attention has been devoted to its impact on rehabilitation, skeletal muscle adaptation, and body composition.

This omission may be particularly important because the biological pathways targeted by immunosuppressive medications overlap extensively with those regulating muscle growth, mitochondrial biogenesis, endothelial function, insulin sensitivity, and metabolic homeostasis. Consequently, rehabilitation outcomes following transplantation occur within a unique physiological environment in which exercise-induced adaptation must coexist with chronic pharmacological modulation of fundamental anabolic and metabolic signaling pathways.

Rather than functioning solely as agents preventing rejection, immunosuppressive drugs may act as powerful modifiers of skeletal muscle plasticity and body composition. Understanding these interactions may be essential for explaining the heterogeneity of functional recovery observed among transplant recipients.

Immunosuppressive therapy represents a central but often underappreciated determinant of post-transplant rehabilitation. Beyond prevention of allograft rejection, these agents influence multiple biological pathways involved in skeletal muscle remodeling, adipose tissue accumulation, endothelial function, mitochondrial metabolism, and exercise responsiveness. The potential rehabilitation-related effects of the major immunosuppressive drug classes, indicating principal mechanisms of action of commonly used immunosuppressive therapies and their potential effects on skeletal muscle physiology, adipose tissue biology, metabolic regulation, vascular function, and exercise-induced adaptation after HTX, are summarized in Table 5.

Importantly, contemporary immunosuppressive strategies vary substantially among transplant centers and individual recipients. Many programs employ steroid-minimization or steroid-withdrawal protocols, whereas others maintain low-dose corticosteroid therapy depending on rejection risk and patient-specific factors. Similarly, conversion between calcineurin inhibitors and mTOR inhibitors may occur in response to renal dysfunction, malignancy risk, metabolic complications, or allograft vasculopathy. Consequently, the effects of immunosuppressive therapy on skeletal muscle adaptation, body composition, and rehabilitation outcomes should not be considered uniform but rather dependent upon individualized immunosuppressive exposure and rejection-risk management.

### 12.2. Skeletal Muscle Plasticity After Heart Transplantation

Successful rehabilitation depends on the capacity of skeletal muscle to undergo adaptive remodeling in response to exercise training. This process requires the coordinated activation of signaling pathways that regulate protein synthesis, mitochondrial biogenesis, angiogenesis, satellite cell activation, and neuromuscular adaptation.

In healthy individuals, endurance exercise stimulates mitochondrial proliferation and oxidative metabolism, whereas resistance exercise promotes muscle hypertrophy by activating anabolic pathways. Together, these mechanisms improve muscle quality, strength, and metabolic efficiency.

Following transplantation, however, these adaptive responses occur in the presence of chronic immunosuppressive therapy. The physiological response to exercise may therefore differ substantially from that observed in healthy populations or even in patients with chronic heart failure. This possibility raises an important yet underexplored question: do transplant recipients experience a form of pharmacologically mediated exercise adaptation?

The answer is likely complex and dependent upon the specific immunosuppressive regimen employed.

### 12.3. Corticosteroids and the Biology of Post-Transplant Sarcopenia

Glucocorticoids remain a cornerstone of many immunosuppressive protocols despite progressive efforts to minimize long-term exposure. Their clinical efficacy is unquestionable; however, their effects on skeletal muscle and metabolism are profound.

Corticosteroids promote protein catabolism through activation of proteolytic pathways and suppression of protein synthesis [125]. Increased expression of muscle-specific ubiquitin ligases accelerates degradation of contractile proteins, while inhibition of anabolic signaling limits recovery of lean tissue. The resulting phenotype resembles accelerated sarcopenia, characterized by reduced muscle mass, diminished strength, and impaired physical performance.

Importantly, glucocorticoid-induced myopathy [126] preferentially affects proximal muscle groups critical for mobility and exercise capacity. This pattern may directly impair participation in rehabilitation programs and contribute to persistent functional limitations despite successful transplantation.

Beyond skeletal muscle, corticosteroids exert substantial effects on adipose tissue distribution. Enhanced adipogenesis, increased appetite, and insulin resistance promote the accumulation of visceral fat. Consequently, corticosteroid exposure may simultaneously reduce muscle mass and increase adiposity, creating conditions favorable for the development of sarcopenic obesity.

These observations suggest that corticosteroids may be a central biological driver of adverse remodeling of body composition after transplantation.

### 12.4. Calcineurin Inhibitors and Metabolic Dysfunction

Calcineurin inhibitors, including tacrolimus and cyclosporine, remain the backbone of contemporary maintenance immunosuppression [127,128]. Their primary mechanism involves inhibition of T-cell activation; however, calcineurin signaling also plays important roles in skeletal muscle biology, mitochondrial function, and vascular regulation.

Experimental studies suggest that calcineurin contributes to muscle fiber specification and oxidative metabolism. Chronic inhibition may therefore influence muscle phenotype and metabolic capacity, although the clinical significance of these effects remains incompletely understood.

More clearly established are the metabolic consequences of calcineurin inhibitor therapy. Hypertension, dyslipidemia, insulin resistance, and post-transplant diabetes are common complications that adversely affect cardiovascular health and exercise performance. Furthermore, endothelial dysfunction associated with calcineurin inhibitor exposure may impair microvascular responsiveness and tissue perfusion.

These effects are particularly relevant within the context of rehabilitation because oxygen delivery and substrate utilization are critical determinants of exercise adaptation. Persistent metabolic abnormalities may therefore limit the ability of transplant recipients to fully benefit from exercise interventions despite restoration of central hemodynamics.

The interaction between calcineurin inhibitor-induced vascular dysfunction and the recovery of endothelial physiology following restoration of pulsatile circulation represents a particularly intriguing area requiring further investigation.

### 12.5. mTOR Inhibitors: A Biological Paradox

Among all immunosuppressive agents, inhibitors of the mammalian target of rapamycin (mTOR) may have the most direct implications for rehabilitation biology.

The mTOR pathway serves as a master regulator of cellular growth, protein synthesis, nutrient sensing, and skeletal muscle hypertrophy [129]. Activation of mTOR signaling is widely recognized as a central mechanism through which resistance exercise stimulates muscle growth and strength development.

Paradoxically, pharmacological inhibition of the same pathway forms the basis of immunosuppressive therapy with sirolimus and everolimus. This creates a unique physiological situation in which one of the principal molecular mediators of exercise-induced adaptation is deliberately suppressed.

From a mechanistic perspective, this raises important questions. To what extent does mTOR inhibition attenuate hypertrophic responses to resistance training? Does chronic suppression of anabolic signaling impair recovery of lean mass following transplantation? Are exercise prescriptions that are effective in non-transplanted populations equally effective in recipients receiving mTOR-based immunosuppression?

At present, these questions remain largely unanswered. Nevertheless, the theoretical implications are substantial. If mTOR inhibition blunts adaptive muscle remodeling, conventional rehabilitation paradigms may require modification to account for altered biological responsiveness.

This represents a major knowledge gap and a potentially important avenue for future translational research.

### 12.6. Immunosuppression, Mitochondrial Function, and Energy Metabolism

Mitochondrial dysfunction has emerged as a central feature of exercise intolerance across numerous cardiovascular and metabolic disorders [130]. Because skeletal muscle oxidative capacity largely depends on mitochondrial quantity and function, factors that influence mitochondrial health may substantially affect rehabilitation outcomes [131].

Several immunosuppressive agents have been implicated in mitochondrial alterations. Experimental studies have demonstrated effects on oxidative phosphorylation, reactive oxygen species production, and cellular energy metabolism. Although the magnitude of these effects in clinical transplantation remains uncertain, they may contribute to persistent reductions in aerobic capacity despite normal graft performance.

This possibility is particularly relevant because improvements in peak oxygen uptake following transplantation frequently remain below age-predicted values. Persistent mitochondrial abnormalities may help explain this discrepancy and reinforce the concept that peripheral limitations continue to dominate exercise performance after transplantation.

### 12.7. Immunosuppression and Adipose Tissue Biology

Traditionally regarded as an inert energy storage compartment, adipose tissue is now recognized as an active endocrine and immune organ capable of influencing systemic metabolism and inflammation.

Immunosuppressive therapy profoundly affects adipose tissue biology [132]. Corticosteroids promote adipocyte differentiation and lipid accumulation, while metabolic disturbances induced by calcineurin inhibitors further facilitate adipose tissue expansion. The resulting increase in visceral adiposity may amplify inflammatory signaling through production of cytokines, adipokines, and other bioactive mediators.

This phenomenon creates a potentially self-reinforcing cycle. Increased adiposity promotes insulin resistance and inflammation, which impair muscle function and physical activity. Reduced activity further exacerbates adipose tissue accumulation and metabolic dysfunction.

Consequently, the metabolic consequences of immunosuppression may extend far beyond simple weight gain and may contribute directly to impaired rehabilitation outcomes.

### 12.8. Immunosuppression as a Modulator of Exercise Responsiveness

A fundamental assumption underlying contemporary rehabilitation is that exercise elicits predictable physiological adaptations. However, this assumption may not fully apply to transplant recipients.

Immunosuppressive therapy influences many of the same molecular pathways activated by exercise, including inflammatory signaling, protein turnover, mitochondrial biogenesis, and cellular growth. Therefore, individual responses to rehabilitation may depend not only on training intensity and adherence but also on the specific immunosuppressive environment in which exercise occurs.

This concept introduces the possibility of “exercise responsiveness phenotypes” among transplant recipients. Patients receiving different immunosuppressive regimens may exhibit distinct patterns of muscle adaptation, body composition remodeling, and metabolic improvement despite participating in similar rehabilitation programs.

Although largely unexplored, this hypothesis could help explain the marked interindividual variability observed in post-transplant recovery and may eventually support more personalized rehabilitation strategies.

**Figure 3:**
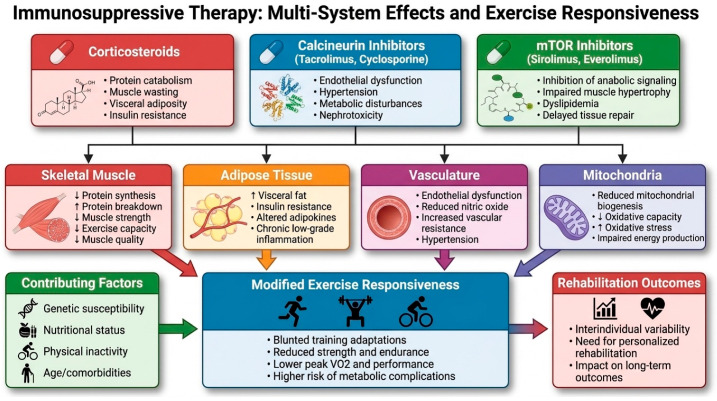
Effects of immunosuppressive therapy on biological pathways involved in rehabilitation after heart transplantation. (Created with FigureLabs by T. Urbanowicz (ID: FL-PUB-20260611-HA7QMM). https://www.figurelabs.ai/ (accessed on 10 June 2026)).

Immunosuppressive agents influence numerous biological pathways involved in skeletal muscle remodeling, adipose tissue accumulation, endothelial function, and mitochondrial metabolism. Consequently, exercise-induced adaptation following transplantation occurs within a unique pharmacologically modified environment that may substantially affect rehabilitation responsiveness, as presented in Figure 3.

### 12.9. Exercise Responsiveness Phenotypes After Heart Transplantation

An emerging concept worthy of future investigation is the existence of exercise responsiveness phenotypes among heart transplant recipients. In non-transplant populations, substantial interindividual variability in adaptation to both aerobic and resistance exercise has been consistently demonstrated. Similar variability is frequently observed following transplantation, where some recipients achieve near-normal functional capacity whereas others exhibit persistent exercise intolerance despite comparable rehabilitation programs. We propose that immunosuppressive therapy may represent one of the principal biological determinants of this variability.

Because corticosteroids, calcineurin inhibitors, and mTOR inhibitors influence pathways regulating protein turnover, mitochondrial function, endothelial biology, and metabolic homeostasis, exercise adaptation may occur within distinct pharmacological environments. Consequently, identical training stimuli may produce different physiological responses according to the prevailing immunosuppressive regimen. Recognition of such exercise responsiveness phenotypes may provide a foundation for individualized rehabilitation strategies in which exercise prescription is adapted not only to clinical characteristics but also to underlying biological responsiveness. Prospective studies integrating exercise testing, body composition analysis, molecular biomarkers, and immunosuppressive exposure are required to determine whether such phenotypes can be identified and clinically exploited.

## 13. Toward an Integrated Immunometabolic Model of Rehabilitation

The traditional view of immunosuppression and rehabilitation treats these domains as largely independent. Immunosuppressive therapy prevents rejection, whereas exercise training improves functional capacity. Emerging evidence suggests that this separation may be artificial.

**Figure 4:**
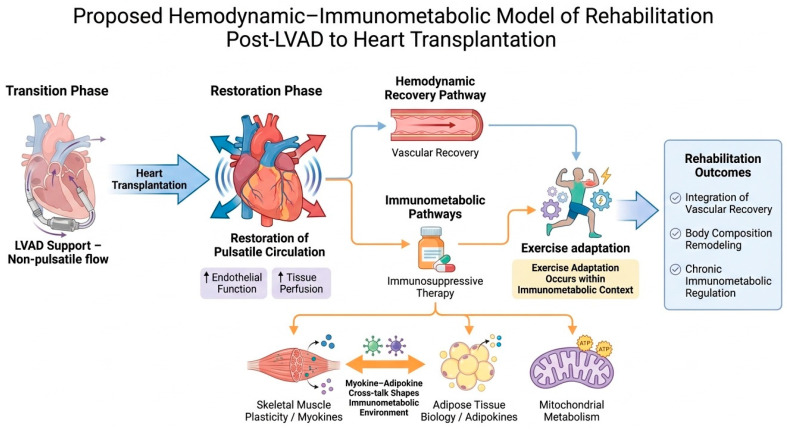
Proposed hemodynamic–immunometabolic model of rehabilitation after transition from LVAD support to heart transplantation. (Created with FigureLabs by T. Urbanowicz (ID: FL-PUB-20260707-Z4C4DZ). https://www.figurelabs.ai/ (accessed on 10 June 2026)).

The central hypothesis of this review is that rehabilitation outcomes after heart transplantation arise from interactions among hemodynamic recovery, body composition remodeling, immunosuppressive therapy, and peripheral tissue adaptation, as presented in Figure 4. Rather than acting independently, these domains form an interconnected biological network that ultimately determines exercise capacity and long-term functional status.

Instead, rehabilitation outcomes appear to arise from a complex interaction among restored hemodynamics, skeletal muscle biology, adipose tissue remodeling, and chronic pharmacological immune modulation. Immunosuppressive therapy shapes the metabolic environment in which exercise adaptation occurs, influences body composition trajectories, and may alter the biological capacity for recovery itself.

Within this framework, exercise training should no longer be viewed merely as a behavioral intervention but rather as a biological stimulus interacting continuously with immunometabolic pathways. Understanding these interactions may represent a critical step toward precision rehabilitation after HTX.

Ultimately, the success of post-transplant rehabilitation may depend not only on restoring pulsatile circulation and preserving graft function but also on optimizing the complex relationship between immunosuppression, body composition, and skeletal muscle adaptation.

Contracting muscle secretes numerous bioactive molecules, collectively termed myokines, that regulate glucose metabolism, inflammation, vascular function, and tissue repair. Healthy skeletal muscle contributes to insulin sensitivity, mitochondrial health, and systemic metabolic homeostasis.

Loss of muscle mass therefore has consequences extending far beyond physical strength. Reduced muscle quantity diminishes whole-body glucose disposal, promotes insulin resistance, and contributes to chronic low-grade inflammation. These effects may interact directly with immunosuppressive therapy, obesity, and endothelial dysfunction.

Conversely, restoration of muscle mass through exercise training may generate benefits that extend beyond functional performance. Improvements in muscle quality may influence inflammatory pathways, metabolic regulation, and vascular health, potentially modifying long-term transplant outcomes.

Increasing evidence suggests that communication between adipose tissue and skeletal muscle plays a central role in regulating metabolic health, inflammation, and exercise adaptation after transplantation. This bidirectional crosstalk is mediated by numerous adipokines and myokines that influence vascular biology, insulin sensitivity, muscle remodeling, and immune regulation. Major signaling molecules derived from adipose tissue and skeletal muscle that may contribute to the regulation of inflammation, metabolism, vascular function, and exercise adaptation in heart transplant recipients are presented in Table 6.

## 14. A New Conceptual Model: From Cachexia to Sarcopenic Obesity

Viewed longitudinally, the LVAD–transplant pathway may be characterized by a progressive evolution of body composition rather than a simple recovery from heart failure.

Patients frequently begin with severe catabolism, muscle wasting, and cachexia during advanced heart failure. LVAD implantation partially reverses this process, often resulting in weight gain that exceeds restoration of lean tissue. Following transplantation, additional metabolic influences—including immunosuppressive therapy, physical inactivity, and altered energy balance—may drive further accumulation of adipose tissue while residual deficits in muscle quality persist.

The result is a continuum extending from cachexia to sarcopenic obesity rather than a return to normal physiological body composition.

This framework provides a potential explanation for why normalization of cardiac function does not necessarily produce normalization of exercise capacity. It also suggests that successful rehabilitation should be evaluated not solely by improvements in peak oxygen consumption or walking distance but by favorable remodeling of skeletal muscle and adipose tissue compartments.

Understanding this trajectory is essential because body composition may represent the biological interface through which hemodynamic recovery, exercise training, and immunosuppressive therapy converge to determine long-term functional outcomes after HTX.

## 15. Immunometabolism, Adipose Tissue–Muscle Crosstalk, and the Biological Basis of Rehabilitation After Heart Transplantation

### 15.1. Rehabilitation as an Immunometabolic Process

Conventional models of cardiac rehabilitation primarily focus on restoring exercise capacity by improving cardiovascular performance and physical conditioning. However, emerging evidence suggests that recovery following HTX cannot be fully understood within this framework alone. Instead, rehabilitation appears to represent a complex immunometabolic process involving dynamic interactions among skeletal muscle, adipose tissue, the vascular endothelium, and the immune system.

This perspective is particularly relevant for transplant recipients because physiological adaptation occurs amid chronic immunosuppression, altered body composition, and persistent low-grade inflammation. Consequently, exercise training influences not only functional capacity but also systemic metabolic and immunological homeostasis.

Within this framework, skeletal muscle and adipose tissue should be viewed as active biological organs that participate in bidirectional communication networks regulating inflammation, metabolism, and vascular function. Rehabilitation may therefore be conceptualized as an intervention targeting an integrated immunometabolic system rather than simply improving aerobic fitness. At present, exercise responsiveness phenotypes should be considered a conceptual framework requiring prospective validation before clinical implementation.

### 15.2. Adipose Tissue as an Active Immune Organ

The traditional view of adipose tissue as a passive energy reservoir has been replaced by recognition of its endocrine and immunological functions [142]. Adipocytes and resident immune cells produce numerous biologically active mediators that influence systemic metabolism, vascular homeostasis, and inflammatory signaling.

Visceral adipose tissue is particularly important because of its high metabolic activity and inflammatory potential. Increased visceral adiposity is associated with elevated production of tumor necrosis factor-α, interleukin-6, monocyte chemoattractant protein-1, leptin, and other pro-inflammatory mediators. These molecules contribute to endothelial dysfunction, insulin resistance, oxidative stress, and vascular remodeling.

The relevance of these mechanisms in transplantation may be substantial. Following transplantation, weight gain and visceral fat accumulation frequently occur as a consequence of corticosteroid exposure, improved appetite, reduced catabolism, and physical inactivity. Although often regarded as benign recovery from chronic illness, excessive adipose tissue expansion may create a persistent inflammatory environment capable of influencing long-term cardiovascular and metabolic outcomes.

Importantly, this process develops despite pharmacological immunosuppression. Indeed, obesity-associated inflammation and alloimmune responses represent distinct biological pathways that may coexist within transplant recipients. Consequently, patients may experience chronic metabolic inflammation even in the absence of clinically significant rejection.

### 15.3. Skeletal Muscle as an Endocrine and Immunoregulatory Organ

Skeletal muscle is increasingly recognized as one of the largest endocrine organs in the human body [143]. Contracting muscle releases numerous signaling molecules, collectively termed myokines, which influence local and systemic physiology [144].

Exercise-induced myokines participate in the regulation of glucose metabolism, lipid oxidation, mitochondrial function, angiogenesis, and inflammatory balance [145]. These effects extend beyond the musculoskeletal system and contribute to whole-body metabolic homeostasis.

Among the most extensively studied myokines is interleukin-6. Although traditionally classified as a pro-inflammatory cytokine, exercise-induced IL-6 appears to exert predominantly anti-inflammatory and metabolic effects, stimulating glucose uptake and promoting production of anti-inflammatory mediators [146,147]. Similarly, irisin has been implicated in adipose tissue browning and energy expenditure, whereas reductions in myostatin signaling may facilitate muscle growth and regeneration.

Loss of skeletal muscle mass therefore has consequences extending far beyond reduced strength. Diminished muscle quantity and quality may reduce myokine production, impair glucose disposal, exacerbate insulin resistance, and contribute to systemic metabolic dysfunction [148].

This concept is particularly relevant in transplant recipients, where persistent sarcopenia may represent not merely a marker of illness but an active contributor to adverse outcomes.

#### Frailty, Myosteatosis, and Muscle Quality

Traditional assessments of body composition have focused primarily on the quantification of muscle mass. However, emerging evidence suggests that muscle quality may be equally important in determining physical performance and clinical outcomes. The concept of myosteatosis, defined as pathological fat infiltration within skeletal muscle, has attracted growing attention as a marker of impaired muscle function and metabolic health.

Myosteatosis may develop as a consequence of chronic heart failure, prolonged inactivity, aging, obesity, and exposure to immunosuppressive therapy [149,150]. Unlike sarcopenia, which reflects loss of muscle mass, myosteatosis represents deterioration of muscle composition and contractile efficiency. Consequently, patients may exhibit apparently preserved muscle mass despite substantial functional impairment.

This distinction is particularly relevant following transplantation. Weight gain and increases in lean body mass do not necessarily indicate restoration of healthy skeletal muscle architecture. Intramuscular lipid accumulation may impair mitochondrial function, reduce insulin sensitivity, and contribute to persistent exercise intolerance.

Frailty provides an additional framework for understanding these abnormalities. Increasingly recognized as a multidimensional syndrome encompassing weakness, reduced physiological reserve, impaired mobility, and vulnerability to stressors, frailty frequently persists despite successful transplantation. Consequently, rehabilitation should target not only restoration of muscle mass but also improvement of muscle quality and reversal of frailty-related deficits.

### 15.4. Adipose Tissue–Muscle Crosstalk in the Post-Transplant State

The interaction between adipose tissue and skeletal muscle represents one of the central mechanisms linking body composition to rehabilitation outcomes.

Under physiological conditions, these tissues engage in continuous endocrine communication that coordinates energy metabolism, inflammatory regulation, and tissue adaptation. However, adverse body composition remodeling may disrupt this balance.

Increased visceral adiposity promotes inflammatory signaling and insulin resistance, impairing muscle protein synthesis and mitochondrial function [151]. Simultaneously, reduced muscle mass diminishes glucose utilization and physical activity, facilitating further adipose tissue accumulation. This creates a self-perpetuating cycle characterized by progressive metabolic dysfunction and declining physical performance.

The coexistence of obesity and sarcopenia may therefore produce a biological state considerably more detrimental than either condition alone. This phenomenon may help explain why some transplant recipients exhibit persistent exercise intolerance despite preserved graft function and apparently successful rehabilitation.

Importantly, immunosuppressive therapy may amplify these interactions by simultaneously promoting adiposity and impairing anabolic signaling.

## 16. Endothelial Function as the Missing Link

The vascular endothelium may represent the critical interface connecting hemodynamics, metabolism, and exercise adaptation.

Endothelial cells respond to mechanical stimuli, inflammatory mediators, metabolic signals, and exercise-induced changes in blood flow [152]. By regulating nitric oxide bioavailability and vascular tone, the endothelium influences skeletal muscle perfusion, insulin sensitivity, and mitochondrial function.

Continuous-flow LVAD support alters endothelial mechanotransduction through reduced pulsatility, whereas transplantation restores physiological pulsatile flow [153]. However, endothelial recovery occurs within an environment shaped by obesity, immunosuppression, and persistent metabolic dysfunction.

Consequently, restoration of pulsatile circulation may not necessarily result in complete normalization of endothelial function. Ongoing inflammatory and metabolic disturbances may continue to impair microvascular responsiveness despite successful transplantation.

This observation provides a potential mechanistic explanation for the persistence of peripheral limitations after restoration of central hemodynamics.

### Cardiac Allograft Vasculopathy: A Potential Immunometabolic Consequence of Adverse Body Composition

Cardiac allograft vasculopathy (CAV) remains one of the leading causes of late morbidity and mortality following HTX. Traditionally regarded as an immune-mediated process driven by chronic endothelial injury and alloimmune activation, CAV is increasingly recognized as a multifactorial disorder involving metabolic, inflammatory, and vascular mechanisms [154,155].

Several components of the adverse immunometabolic phenotype described in this review overlap with established risk factors for CAV development. Visceral adiposity promotes chronic low-grade inflammation, insulin resistance, oxidative stress, and endothelial dysfunction, all of which may contribute to vascular remodeling within the transplanted heart [156]. Similarly, persistent skeletal muscle dysfunction and reduced physical activity may exacerbate metabolic abnormalities that adversely affect vascular health.

The vascular endothelium may represent the biological interface linking body composition and allograft outcomes [157]. Endothelial dysfunction arising from obesity, metabolic syndrome, and chronic inflammation may amplify the endothelial injury central to CAV pathogenesis [158]. Consequently, adverse body composition may influence not only rehabilitation outcomes but potentially long-term graft survival [159].

Although direct evidence linking sarcopenic obesity and myosteatosis to CAV remains limited, the convergence of these biological pathways suggests an important area for future investigation. Understanding whether rehabilitation-induced improvements in body composition can favorably influence CAV risk may represent a particularly attractive translational research opportunity.

An intriguing possibility is that adverse body composition may influence not only rehabilitation outcomes but also the biological processes governing long-term graft health. The coexistence of visceral adiposity, endothelial dysfunction, insulin resistance, and chronic low-grade inflammation creates a systemic environment that closely parallels pathways implicated in the development of cardiac allograft vasculopathy. Although direct causal relationships remain unproven, it is conceivable that body composition remodeling represents a modifiable determinant of vascular health within the transplanted heart. If confirmed, rehabilitation-induced improvements in skeletal muscle quality and visceral adiposity could extend beyond enhancement of functional capacity and potentially contribute to long-term graft preservation. This hypothesis highlights the need to view rehabilitation not solely as a supportive intervention but as a potential modifier of transplant biology itself.

## 17. Exercise as an Immunometabolic Intervention

Exercise training exerts effects extending far beyond improvements in aerobic capacity. Contemporary evidence suggests that exercise should be viewed as a potent immunometabolic intervention capable of simultaneously influencing skeletal muscle, adipose tissue, endothelial function, and inflammatory regulation [160].

Aerobic exercise improves mitochondrial function, insulin sensitivity, and endothelial responsiveness while reducing visceral adiposity [161,162]. Resistance exercise stimulates muscle protein synthesis, enhances neuromuscular performance, and counteracts sarcopenia [163,164]. Combined training may therefore target multiple components of the adverse immunometabolic phenotype observed after transplantation.

Importantly, exercise may also modify inflammatory signaling. Reductions in adipose tissue mass decrease production of pro-inflammatory adipokines, whereas increased skeletal muscle activity enhances release of beneficial myokines. These adaptations collectively promote a shift toward a more favorable metabolic and inflammatory profile.

From this perspective, rehabilitation represents not merely a means of improving exercise capacity but a strategy for modifying the biological environment in which long-term transplant outcomes develop.

**Figure 5:**
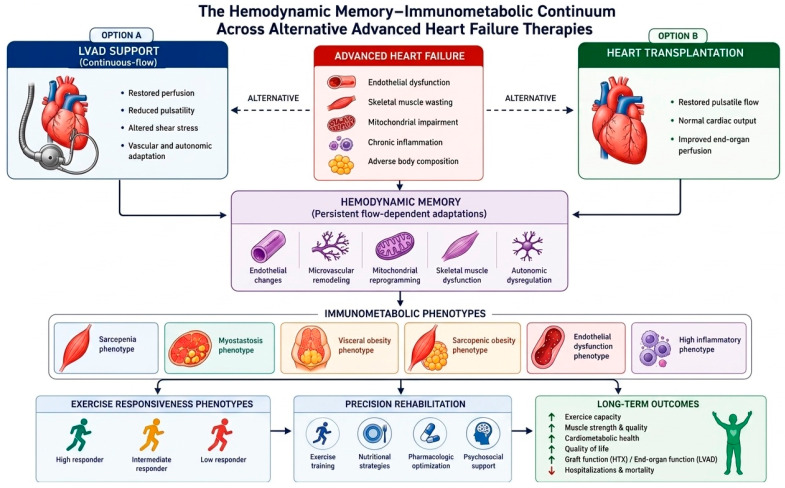
The Hemodynamic Memory–Immunometabolic Continuum: proposed biological pathway linking advanced heart failure, continuous-flow LVAD support, and long-term rehabilitation outcomes after heart transplantation. (Created with FigureLabs by T. Urbanowicz (ID: FL-PUB-20260611-E3WTBI). https://www.figurelabs.ai/ (accessed on 10 June 2026)).

The evidence reviewed in this manuscript suggests that rehabilitation outcomes following heart transplantation emerge from the interaction of multiple biological processes extending beyond restoration of cardiac function alone. We propose that exposure to advanced heart failure and continuous-flow mechanical support may leave a persistent biological imprint, termed hemodynamic memory, which subsequently interacts with immunosuppressive therapy, body composition remodeling, and peripheral tissue adaptation after transplantation. This integrated perspective provides a conceptual framework linking vascular recovery, immunometabolic regulation, and exercise responsiveness, and may help explain the marked heterogeneity observed in post-transplant functional outcomes. The proposed hemodynamic memory–immunometabolic continuum is summarized in Figure 5.

## 18. Clinical Implications: Toward Precision Rehabilitation After Heart Transplantation

### 18.1. Limitations of Current Rehabilitation Models

Current rehabilitation programs for heart transplant recipients are largely adapted from protocols developed for patients with coronary artery disease or chronic heart failure [56]. Although these approaches improve functional capacity, they may inadequately address the unique physiological challenges associated with transplantation.

Most contemporary programs focus primarily on aerobic performance, exercise tolerance, and quality-of-life outcomes [165,166,167]. Relatively little attention is devoted to body composition, immunometabolic status, or the potential influence of specific immunosuppressive regimens on exercise adaptation.

Recognition of the heterogeneity of transplant recipients suggests that rehabilitation strategies should move beyond a one-size-fits-all approach. Distinct body composition, vascular, metabolic, and immunological phenotypes may require individualized therapeutic approaches to optimize recovery. A proposed conceptual phenotype-driven approach integrating body composition, vascular function, inflammatory status, and immunosuppressive exposure to guide individualized rehabilitation interventions and improve long-term functional outcomes is outlined in Table 7.

This approach may overlook important biological determinants of recovery.

### 18.2. Beyond Peak Oxygen Consumption

Peak oxygen uptake remains the most widely utilized measure of functional capacity following transplantation [178]. However, reliance on peak VO_2_ alone may provide an incomplete assessment of recovery.

Patients with similar peak VO_2_ values may differ substantially in muscle mass, visceral adiposity, metabolic health, and inflammatory status [179]. Consequently, physiological recovery cannot be fully captured by aerobic performance measures alone.

Future rehabilitation assessment may require incorporation of body composition analysis, muscle strength testing, metabolic profiling, and biomarkers of inflammation alongside traditional exercise parameters.

Such an approach would provide a more comprehensive understanding of patient status and facilitate individualized intervention strategies.

### 18.3. Personalizing Rehabilitation According to Biological Phenotype

Growing evidence suggests that transplant recipients represent a heterogeneous population characterized by distinct physiological phenotypes.

Some patients exhibit predominant sarcopenia, whereas others develop obesity or sarcopenic obesity. Some experience significant metabolic dysfunction despite preserved physical performance, whereas others maintain favorable metabolic profiles despite reduced exercise capacity.

Recognition of these differences may support development of precision rehabilitation strategies.

For example, patients with sarcopenia may benefit from greater emphasis on resistance training and nutritional support. Individuals with visceral obesity may require interventions targeting adipose tissue reduction and metabolic health. Recipients receiving mTOR-based immunosuppression may potentially require alternative strategies to optimize muscle adaptation.

Although such approaches remain largely theoretical, they represent a logical extension of precision medicine principles into transplant rehabilitation.

### 18.4. Future Directions and Research Priorities

Several critical knowledge gaps remain.

First, the extent to which restoration of pulsatile circulation reverses vascular and skeletal muscle adaptations induced by continuous-flow LVAD support remains poorly understood. Longitudinal studies examining endothelial function, muscle perfusion, and mitochondrial recovery across the LVAD–transplant transition are needed.

Second, the interaction between specific immunosuppressive regimens and exercise-induced adaptation requires systematic investigation. Particular attention should be directed toward the effects of mTOR inhibition on muscle hypertrophy and training responsiveness.

Third, comprehensive body composition phenotyping should be incorporated into future rehabilitation studies. Distinguishing between obesity, sarcopenia, and sarcopenic obesity may improve risk stratification and therapeutic targeting.

Finally, mechanistic studies integrating hemodynamic, metabolic, immunological, and musculoskeletal variables are needed to establish causal pathways linking transplantation, body composition, and functional recovery.

## 19. Conclusions

The transition from continuous-flow LVAD support to heart transplantation represents a unique physiological model characterized by restoration of pulsatile circulation, profound alterations in body composition, and lifelong exposure to immunosuppressive therapy. While transplantation effectively corrects central hemodynamic impairment, recovery of functional capacity depends largely on peripheral adaptations involving skeletal muscle, adipose tissue, microvascular function, and metabolic regulation.

Emerging evidence suggests that rehabilitation outcomes are governed by a complex interaction among hemodynamic recovery, immunosuppressive therapy, and immunometabolic remodeling. Skeletal muscle and adipose tissue function as active endocrine organs whose reciprocal communication influences inflammation, metabolism, and exercise responsiveness. Simultaneously, immunosuppressive agents modify many of the biological pathways required for physiological adaptation to exercise.

We propose a novel hemodynamic–immunometabolic framework in which restoration of pulsatile circulation provides the physiological substrate for recovery, while persistent vascular adaptation, body composition remodeling, skeletal muscle plasticity, and chronic immunosuppressive exposure collectively determine the extent to which recovery can be realized. Within this framework, the concept of hemodynamic memory is introduced as a potential mechanism linking prior exposure to advanced heart failure and continuous-flow support with subsequent heterogeneity of rehabilitation outcomes. We further propose that immunosuppressive therapy may contribute to the emergence of distinct exercise responsiveness phenotypes, thereby influencing the biological effectiveness of rehabilitation interventions. Together, these concepts extend current rehabilitation paradigms beyond restoration of cardiac output and establish a foundation for precision rehabilitation strategies after heart transplantation.

The proposed hemodynamic–immunometabolic model should be viewed as a conceptual framework intended to integrate existing observations and generate future research hypotheses. Prospective studies incorporating vascular phenotyping, body composition assessment, skeletal muscle biology, and rehabilitation outcomes will be required to determine whether these proposed mechanisms contribute meaningfully to functional recovery after heart transplantation.

## Figures and Tables

**Table 1:** Physiological characteristics across the heart failure–LVAD–heart transplantation continuum.

Domain	Advanced Heart Failure	Continuous-Flow LVAD	Heart Transplantation
Cardiac output	Severely reduced	Restored	Normalized
Pulsatility	Reduced	Markedly reduced/non-physiological	Physiological
Endothelial function	Impaired [30]	Partially improved but abnormal [31]	Improved but often incompletely restored [32]
Microvascular perfusion	Reduced [33]	Improved [34]	Further improved [35]
Baroreceptor function	Impaired [36]	Persistently altered [37]	Partial recovery [38]
Skeletal muscle perfusion	Reduced [39]	Improved but suboptimal [40]	Improved [41]
Mitochondrial function	Impaired [42]	Partial recovery [43]	Incomplete normalization [44]
Exercise capacity	Severely limited [45]	Moderately improved [46]	Significantly improved but often below predicted values [47]
Inflammatory burden	High [48]	Reduced but persistent [49]	Modified by immunosuppression [50]
Body composition	Cachexia/sarcopenia [51]	Fat gain > muscle gain [25]	Obesity/sarcopenic obesity common [52]
Dominant limitation	Cardiac failure	Peripheral dysfunction	Immunometabolic and peripheral dysfunction

Abbreviation: LVAD, left ventricular assist device.

**Table 2:** Established guidelines and recommendations for rehabilitation following HTX/LVAD therapy.

Domain	Established Evidence	Current Guideline Position	Knowledge Gap	Contribution of Present Review
LVAD rehabilitation	Improves functional capacity	Recommended	Mechanisms unclear	Focus on vascular adaptation
HTx rehabilitation	Improves VO_2_peak	Recommended	Variable response	Biological determinants of responsiveness
Sarcopenia/frailty	Associated with prognosis	Increasingly recognized	Understudied after LVAD-HTx transition	Integrative body composition framework
Immunosuppression	Metabolic side effects known	Not integrated into rehabilitation planning	Impact on adaptation poorly understood	Exercise-responsiveness model
Pulsatility	Biological relevance established	Not addressed in rehabilitation guidelines	Long-term consequences uncertain	Hemodynamic-memory hypothesis

Abbreviations: HTX—heart transplantation, LVAD—left ventricular assist device, and VO_2_—volume of oxygen consumption.

**Table 3:** Hemodynamic consequences of continuous-flow LVAD support and restoration of pulsatility following heart transplantation.

Biological System	Continuous-Flow LVAD	Restored Pulsatility After HTX	Potential Rehabilitation Consequences	References
Endothelium	Reduced shear stress signaling	Physiological mechanotransduction restored	Improved vascular responsiveness	[31,65]
Nitric oxide bioavailability	Reduced	Increased	Better skeletal muscle perfusion	[66,67]
Arterial compliance	Decreased	Improved	Enhanced exercise hemodynamics	[68,69]
Microcirculation	Capillary recruitment abnormalities	Improved capillary regulation	Better oxygen extraction	[70,71]
Autonomic regulation	Blunted baroreceptor activation	Partial normalization	Improved exercise tolerance	[72,73]

Abbreviations: HTX, heart transplantation; LVAD, left ventricular assist device.

**Table 4:** Preferred assessment for body composition phenotypes.

Phenotype	Preferred Assessment	References
Sarcopenia	DXA, CT, MRI, BIA	[103,104,105]
Myosteatosis	CT, MRI, ultrasound	[106,107,108]
Visceral obesity	CT, MRI, DXA, BIA	[109,110]
Sarcopenic obesity	Combined body composition + strength assessment	[111,112]

Abbreviations: BIA—bioelectrical impedance analysis; CT—computed tomography; DXA—Dual-energy X-ray absorptiometry; MRI—magnetic resonance imaging.

**Table 5:** Effects of major immunosuppressive agents on rehabilitation, body composition, and exercise adaptation.

Drug Class	Principal Mechanism	Skeletal Muscle Effects	Adipose Tissue Effects	Vascular/Metabolic Effects	Potential Impact on Rehabilitation	References
Corticosteroids	Broad immunosuppression	Protein catabolism, myopathy, reduced strength	Increased visceral adiposity	Insulin resistance, diabetes risk	Reduced exercise responsiveness, sarcopenic obesity	[115,116]
Calcineurin inhibitors (Tacrolimus/Cyclosporine)	T-cell inhibition	Potential alterations in oxidative metabolism	Indirect fat accumulation	Hypertension, endothelial dysfunction, diabetes	Impaired aerobic adaptation	[117,118]
mTOR inhibitors (Sirolimus/Everolimus)	Inhibition of mTOR signaling	Potential suppression of muscle hypertrophy and protein synthesis	Variable effects	Dyslipidemia, metabolic alterations	Possible blunting of resistance training adaptations	[119,120]
Antimetabolites (Mycophenolate)	Inhibition of lymphocyte proliferation	Minimal direct effects	Limited evidence	Generally metabolically neutral	Limited direct impact	[121,122]
Combination therapy	Multiple pathways	Cumulative effects	Enhanced obesity risk	Complex metabolic phenotype	Heterogeneous rehabilitation response	[123,124]

Abbreviation: mTOR, mammalian target of rapamycin.

**Table 6:** Adipokines and myokines potentially involved in post-transplant immunometabolic remodeling.

Mediator	Primary Source	Major Actions	Potential Relevance After HTX	References
Leptin	Adipose tissue	Appetite regulation, inflammation	Promotes obesity-related inflammation and post-transplant diabetes	[133]
Adiponectin	Adipose tissue	Anti-inflammatory, insulin-sensitizing	Potentially protective against metabolic dysfunction	[134]
TNF-α	Adipose tissue/macrophages	Catabolism, insulin resistance	Contributes to muscle dysfunction	[135]
IL-6 (adipose-derived)	Visceral adipose tissue	Chronic inflammation	Endothelial dysfunction, metabolic impairment	[136]
IL-6 (exercise-derived)	Skeletal muscle	Anti-inflammatory, glucose regulation	Beneficial exercise adaptation	[137]
Irisin	Skeletal muscle	Browning of adipose tissue	Improved metabolic profile	[138]
Myostatin	Skeletal muscle	Inhibits muscle growth	Promotes sarcopenia	[139]
IGF-1	Skeletal muscle/liver	Anabolic signaling	Supports muscle recovery	[140]
VEGF	Skeletal muscle/endothelium	Angiogenesis	Improves perfusion and adaptation	[141]

Abbreviations: HTX, heart transplantation; IGF-1, insulin-like growth factor 1; IL-6, interleukin-6; TNF-α, tumor necrosis factor alpha; VEGF, vascular endothelial growth factor.

**Table 7:** Proposed precision rehabilitation framework following LVAD support and heart transplantation.

Phenotype	Clinical Characteristics	Biological Features	Rehabilitation Priorities	References
Persistent sarcopenia	Low muscle mass, weakness	Impaired anabolic signaling, reduced myokine activity	Resistance training, protein supplementation	[168,169,170]
Visceral obesity	Increased abdominal adiposity	Adipokine dysregulation, insulin resistance	Aerobic exercise, weight reduction	[171,172]
Sarcopenic obesity	Excess fat and low muscle quality	Combined inflammatory and anabolic impairment	Combined aerobic-resistance training	[173,174]
Endothelial dysfunction phenotype	Reduced exercise tolerance despite preserved graft function	Impaired NO signaling, microvascular dysfunction	Aerobic interval training, vascular-targeted interventions	[4,175]
mTOR inhibitor phenotype	Reduced hypertrophic response	Suppressed anabolic signaling	Individualized resistance exercise prescription	[176]
High inflammatory phenotype	Elevated metabolic dysfunction	Adipose-muscle inflammatory crosstalk	Multimodal exercise and nutritional intervention	[177]

Abbreviations: mTOR, mammalian target of rapamycin; NO, nitric oxide.

## Data Availability

There were no data created supporting the publication.

## Abbreviations

The following abbreviations are used in this manuscript:

BIA	Bioelectrical Impedance Analysis
BMI	Body Mass Index
CNI	Calcineurin Inhibitor
CT	Computed Tomography
DXA	Dual-Energy X-ray Absorptiometry
EAPC	European Association of Preventive Cardiology
ECMO	Extracorporeal Membrane Oxygenation
eNOS	Endothelial Nitric Oxide Synthase
ESOT	European Society for Organ Transplantation
EWGSOP2	European Working Group on Sarcopenia in Older People 2
HFA	Heart Failure Association
HF	Heart Failure
HTX	Heart Transplantation
IABP	Intra-Aortic Balloon Pump
ISHLT	International Society for Heart and Lung Transplantation
LVAD	Left Ventricular Assist Device
MCS	Mechanical Circulatory Support
MeSH	Medical Subject Headings
MRI	Magnetic Resonance Imaging
mTOR	Mammalian Target of Rapamycin
NO	Nitric Oxide
VA-ECMO	Venoarterial Extracorporeal Membrane Oxygenation
VEGF	Vascular Endothelial Growth Factor
VO_2_	Oxygen Consumption
VO_2_peak	Peak Oxygen Uptake
